# Effects of fipronil bait pellets on two cricetid species: Potential implications for plague mitigation and wildlife conservation

**DOI:** 10.1016/j.ijppaw.2026.101239

**Published:** 2026-05-22

**Authors:** David A. Eads, Marc R. Matchett, Travis M. Livieri, Richard A. Bowen, Airn E. Hartwig, Stephanie Porter, Mary L. Wright, Jason Fly, Madisen Hartlaub, Phillip Dobesh, Paul Roghair, Eddie Childers, John P. Hughes, Michelle L. Hladik, Gregory P. Dooley, Brian J. Smith, Rachel A. LaCasse, Kristy Bly, Dean E. Biggins

**Affiliations:** a U.S. Geological Survey, Fort Collins Science Center, Fort Collins, CO, 80526, USA; b Eads Ecological Services LLC, Longmont, CO, 80504, USA; c U.S. Fish and Wildlife Service, Charles M. Russell National Wildlife Refuge, Lewistown, MT, 59457, USA; d Prairie Wildlife Research, Stevens Point, WI, 54481, USA; e Animal Reproduction and Biotechnology Laboratory, Colorado State University, Fort Collins, CO, 80523, USA; f U.S. Fish and Wildlife Service, National Black-footed Ferret Conservation Center, Carr, CO, 80612, USA; g National Park Service, Badlands National Park, Interior, SD, 57750, USA; h U.S. Forest Service, Wall Ranger District, Wall, SD, 57790, USA; i U.S. Geological Survey, California Water Science Center, Sacramento, CA, 95819, USA; j College of Veterinary Medicine and Biomedical Sciences, Colorado State University, Fort Collins, CO, 80523, USA; k National Institute of Allergy and Infectious Diseases, National Institute of Health, Rocky Mountain Laboratories, Hamilton, MT, 59840, USA; l World Wildlife Fund, Northern Great Plains Program, Bozeman, MT, 59715, USA

**Keywords:** Cricetid, Fipronil, Flea, Mouse, Plague, Pulicide

## Abstract

We evaluated the effects of fipronil bait pellets on two cricetids that commonly occupy colonies of black-tailed prairie dogs (*Cynomys ludovicianus*; BTPDs): western deer mice (*Peromyscus sonoriensis*) and northern grasshopper mice (*Onychomys leucogaster*). In one experiment, bait pellets (0.96 mg fipronil/bait) were applied at 75 baits/ha to three 1.44-ha plots on a BTPD colony. Mouse abundance declined by 70% from before to 6-10 d after treatment. In a second experiment, bait pellets (0.46 or 1.52 mg fipronil/bait) were applied at 125 baits/ha to four plots (0.85-1.86 ha) on two BTPD colonies; two non-treated plots were baselines (1.09 and 2.06 ha). From before to 11-15 d after treatment, mouse abundance declined by 51%- 67% on the treated plots vs. a decline of 9% on the non-treated plots. Mouse survival from before to 11-15 d after treatment was 51% lower on the treated plots. In a third experiment, bait pellets (0.84 mg fipronil/bait) were applied at 125 baits/acre on two 1.44-ha plots on a BTPD colony; two 1.44-ha non-treated plots were baselines. Mouse survival from before to 30-44 d after treatment was 45% lower on the treated plots; the abundance of deer mice on the treated plots remained similar from before to 30-44 d after treatment, perhaps due to juvenile recruitment and/or immigration. In a laboratory experiment, 33 deer mice offered one bait pellet (0.86 mg fipronil/bait) consumed 27% of their bait, on average (range = 0-100%). Over 3 d, deer mouse mortality was estimated at 53%; mortality increased with fipronil dose, which averaged 11 mg fipronil/kg body mass (range = 3-46 mg/kg). Brain samples were available from 31 deer mice; all tested positive for fipronil sulfone, the primary mammalian metabolite of fipronil, at 19 to 61,205 ng fipronil sulfone/g. Additional experiments could determine if these findings scale up to larger landscapes.

## Highlights

• Flea control is a common method of plague mitigation in rodents
• Some flea control tools may have toxic effects on hosts
• We tested the effects of fipronil bait pellets in deer mice and grasshopper mice
• Baits with 0.40-1.30 mg fipronil/bait pellet reduced mouse survival and abundance
• Field experiments could determine if these findings scale to larger landscapes

## Graphical abstract

**Figure:**
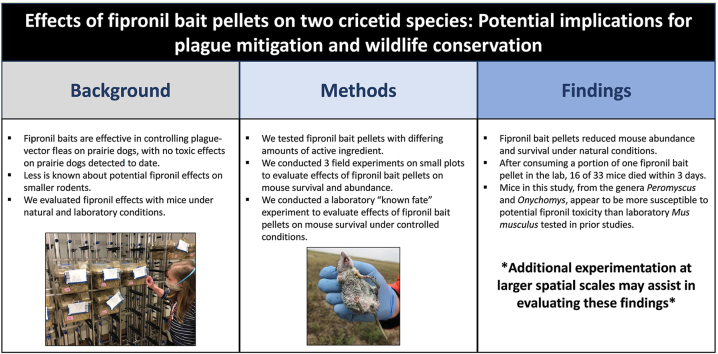

## 1 Introduction

Vector-borne diseases pose a threat to humans and wildlife nearly worldwide. For mitigation purposes, “vector control options that are cost-effective and socially, culturally, and environmentally acceptable are needed” (CDC, 2020:6). Here, we concentrate on vector control to mitigate sylvatic plague, a zoonotic disease caused by the primarily flea-borne bacterium *Yersinia pestis*. Plague is notorious for causing pandemics among humans, including the “Black Death” of medieval Europe. Although still a disease of humans, *Y. pestis* contemporarily associates with wild mammals and their fleas (Gage and Kosoy, 2005). The pathogen is increasingly recognized as an ecosystem transformer (Eads and Biggins, 2015) with meaningful ecological and conservation implications (Zeppelini et al., 2016; Eads et al., 2022).

A variety of tools are available for flea control and plague mitigation, including fipronil, a GABA_A_ receptor antagonist (Hainzl et al., 1998) that has been incorporated into edible baits as a systemic pulicide (i.e., flea-killing agent). Adult fleas that imbibe blood from a host that consumed fipronil bait can be killed, and larval fleas interacting with or feeding on feces from treated hosts can be killed (Poché et al., 2017; Eads et al., 2023). In this study, we concentrated on fipronil bait pellets formulated for flea control and plague mitigation in prairie dogs (*Cynomys* spp., PDs) in western North America (Eads et al., 2021; Matchett et al., 2023).

The fipronil bait pellets were created from inexpensive and readily available food grade ingredients, using simple formulation steps, small amounts of fipronil, and mass production capabilities for making bait pellets (Corro et al., 2017). The bait pellets are typically distributed along transects at a rate of 125 bait pellets/ha (Eads et al., 2021; Matchett et al., 2023). Each pellet is a round, 1.25-cm diameter, blue (food dye) pellet weighing ∼1.25 g. Initial dosing targeted a 1 kg adult PD (Koford, 1958) at 1% of the LD_50_ of technical grade fipronil in laboratory mice (*Mus musculus*; LD_50_ = ∼95 mg fipronil/kg; Connelly, 2011; Gibbons et al., 2015). Compounding for initial testing resulted in ∼0.84 mg fipronil/bait pellet (Eads et al., 2021).

Fipronil bait pellets have been tested for flea control with BTPDs in South Dakota and Montana, and Gunnison's PDs (*Cynomys gunnisoni*, GPDs) in Arizona. In most cases, PD flea-burdens were reduced by 85-100% for at least 12 mo, and for 24 mo in some cases. Impacts of plague on PDs would presumably be reduced by the degree and duration of flea control observed (Biggins et al., 2010). Moreover, relative to other existing methods, fipronil bait pellets may reduce the economic costs of plague mitigation on PD colonies by 80% or more (Eads et al., 2021; Matchett et al., 2023).

With any plague mitigation tool, potential unintended effects on target and non-target species require careful evaluation. Laboratory studies have documented deleterious effects of fipronil and fipronil sulfone (the primary mammalian metabolite) on some vertebrates; however, “effective doses have not typically been matched to realistic field exposure conditions” (Gibbons et al., 2015). Fipronil bait pellets provided effective flea control in PDs and adverse effects in PDs have not been observed in previous studies (Eads et al., 2021; Matchett et al., 2023). Effects on other, sympatric species may depend on taxonomy, body mass, fipronil dosing, season of treatment, animal space use, and the propensity of different species and individuals to find, consume, or avoid the bait pellets; potential bait caching of bait pellets by some rodents may be influential.

After fipronil bait pellets are distributed on a PD colony in the morning, some pellets will remain on the landscape into the night, perhaps over multiple days and nights (e.g., as found with other edible baits; Tripp et al., 2014). Into the night, remaining bait pellets are available to more nocturnal species, including some cricetid mice. There is evidence to suggest fipronil, at some doses, may negatively impact some species of mice, for example by causing genotoxic and mutagenic effects, oxidative stress, infertility, altered emotional, cognitive, parental, or exploratory behaviors, and even death (Gunasekara et al., 2007; Gibbons et al., 2015; Wang et al., 2016; Singh et al., 2021). Such effects, if occurring under field treatment regimens, may reduce the survival and abundance of cricetids.

We evaluated the effects of fipronil bait pellets on the survival and abundance of two cricetid species that associate with PDs throughout much of their ranges: western deer mice (*Peromyscus sonoriensis*, DM) and northern grasshopper mice (*Onychomys leucogaster*, GM; Bron et al., 2018, 2021). Theoretically, one bait pellet containing 0.84 mg fipronil would dose 44% of the laboratory mouse LD_50_ for a 20 g adult DM (vs. ∼1% for a ∼1 kg adult BTPD). Three bait pellets with 0.84 mg fipronil/pellet would represent 133% of the laboratory mouse oral LD_50_. For a 30 g adult GM, one or three bait pellets would represent 29% or 88% of the laboratory mouse oral LD_50_.

We conducted three field experiments to evaluate potential effects of fipronil bait pellets, at varying fipronil concentrations and application rates, on the survival and abundance of DM and GM, with treatments and sampling taking place on small experimental plots established on BTPD colonies. We also conducted a laboratory experiment to evaluate effects of fipronil bait pellets on DM survival. We hypothesized that fipronil bait pellets may kill some mice under natural and laboratory conditions, and mortality would increase with fipronil dosing in captivity.

## 2 Materials and methods

### 2.1 Field experiment study areas

We studied DM and GM within three adjacent, interrelated management areas: Buffalo Gap National Grassland (43°45′N, 102°03′W), including Conata Basin (43°45′N, 102°11′W), and Badlands National Park (43°48′N, 102°07′W), South Dakota, USA (Table 1; Eads et al., 2026). The Buffalo Gap National Grassland is administered by the U.S. Forest Service. Badlands National Park is administered by the National Park Service (NPS). Predominant vegetation included western wheatgrass (*Elymus smithii*), blue grama (*Bouteloua gracilis*), buffalo grass (*Bouteloua dactyloides*), pricklypear cactus (*Opuntia polyacantha*), various species of forbs, and (in some years/areas) invasive yellow sweet clover (*Melilotus officinalis*; Livieri and Anderson, 2012). Primary land uses included cattle grazing (outside Badlands National Park) and recreation (in portions of all areas; Livieri and Anderson, 2012).

**Table 1:** Summary of field experiments evaluating the effects of fipronil bait pellets on two cricetids occupying colonies of black-tailed prairie dogs (*Cynomysludovicianus*) in South Dakota, USA: western deer mice (*Peromyscus sonoriensis*) and northern grasshopper mice (*Onychomys leucogaster*). Experiments were conducted in four years across three prairie dog colonies of varying size under before-after-control-impact (BACI) or before-after (BA) study designs. Numbers (*n*) of plots, plots sizes (ha), treatment buffers (m), distances between plots (m), capture grid sizes (ha), bait pellets applied (per ha), fipronil concentrations (mg fipronil/bait pellet), and the periods of field sampling (d before or after treatment) are included. Nominal concentrations of fipronil are reported below the table (LaBarbera et al., 2026).

											Sampling periods	
Year	Colony	Colony size	Design	*n* plots	Plot sizes	Treatment buffers	Distance between plots	Capture grid size	Bait pellets applied	Fipronil/bait pellet	Before treatment	After treatment
2018	South Exclosure	452 ha	BACI	2 treatment/2 control	1.44 ha	50 m	>650 m	0.81 ha	125/ha	0.84 mg	4-1 d before	30-44 d after
2022	Conata West	395 ha	BA	3 treatment	1.44 ha	50 m	260-380 m	0.81 ha	75/ha	0.96 mg	6-1 d before	6-10 d after
2023	Pinnacles	63 ha	BACI	2 treatment/1 control	1.66 to 2.06 ha	20 m	≥150 m	0.81 ha	125/ha	0.46 and 1.52 mg	3-1 d before	11-15 d after
2023	Prairie Wind South	27 ha	BACI	2 treatment/1 control	0.85 to 1.74 ha	20 m	≥75 m	0.81 ha	125/ha	0.46 and 1.52 mg	3-1 d before	11-15 d after

Fipronil nominal concentrations in individual ∼1.25 g (1250 mg) bait pellets were 0.46 mg = 0.0368%, 0.84 mg = 0.0672%, 0.96 mg = 0.0768%, and 1.52 mg = 0.1216%.

### 2.2 Field experiment designs, treatments, and sampling

The first field experiment (July-September 2018) and third field experiment (July-August 2023) involved before-after-control-impact (BACI) study designs with sampling before and after treatments on replicate treatment (impact) and non-treated (control) sites paired ecologically by similar physiographic and biotic features within BTPD colonies (i.e., a BACI paired design with multiple sites; *sensu* Smith, 2002). The second field experiment (July-August 2022) involved a before-after (BA) design with sampling on treatment sites in the central portion of a BTPD colony before and after treatments (Table 1).

Impact assessments assume all treated and non-treated sites within a pair are statistically independent, and experimental pairs are independent (Smith, 2002). Otherwise, pseudoreplication is influential (Hurlbert, 1984; Johnson, 2002). In our case, we did not detect DM or GM movements between plots or pairs on the same colony in a given year, suggesting the mice were not shifting among plots as pseudoreplicates (Johnson, 2002). In addition, comparisons may have been facilitated by site selections based on ecological similarities. For example, predator communities indexed by opportunistic observations appeared similar.

The study sites were originally established to evaluate effects of fipronil bait pellets (known as "FipBits") on BTPD flea parasitism, body condition, abundance, and survival (Eads et al., 2021). We were also interested in potential effects of treatments on DM and GM. We studied these small mammals on 0.81-ha trapping grids (Krebs et al., 2011) centered on the BTPD sites, with 100 traps/plot, at 10 m spacing (Caro, 2002) between neighboring Sherman traps (123 traps/ha; HB Sherman Inc., Tallahassee, FL, USA; 7.62 × 7.62 × 25.4 cm non-folding and 8 × 9 × 23 cm folding). Throughout this project, mean recapture distances were ∼30 m for DM and ∼50 m for GM. Applying these distances as buffers to each side of the 0.81-ha trapping grids yielded effective trapping areas of ∼2.25 ha and 3.61 ha, respectively.

In 2018, we conducted a BACI experiment on the South Exclosure, a 452-ha BTPD colony in the Conata Basin (Table 1). The experiment included two pairs, each with a non-treated site and a treatment site (1.44 ha each). The pairs were split between northern and southern portions of the colony ∼3 km apart, with >650 m between the edges of paired plots. Fipronil bait pellets (0.84 mg fipronil/bait pellet) were applied in early August (on foot using gloved hands) to the 1.44-ha plots (and 50-m buffers) at 125 bait pellets/ha on grid transects (Eads et al., 2021). Trapping was completed over four nights before treatments and three nights from 30 to 44 d after treatments, in the latter period with 2-8 nights between trapping on some plots (i.e., non-consecutive trapping nights). Paired non-treated and treatment plots were trapped on the same nights.

In 2022, we conducted a BA experiment on a portion of Conata West, estimated in 2021 as 395 ha of BTPD-occupied habitat in the Conata Basin (Table 1). This experiment included three treatment sites, each 1.44 ha in size and separated from the nearest neighbor, edge-to-edge, by ∼260-380 m. Fipronil bait pellets (0.96 mg fipronil/bait pellet, distributed evenly among the plots) were applied (on foot using gloved hands) in early August to all three 1.44 ha plots (and 50 m buffers) at 75 bait pellets/ha, in this case to test a reduced baiting density (vs. 125 bait pellets/ha in 2018). Trapping was completed on all three plots, on consecutive nights, for six nights just before treatments and five nights from 6 to 10 d after treatments.

In 2023, we conducted two replicate experiments on separate BTPD colonies, Pinnacles and Prairie Wind South in Badlands National Park, estimated in 2022 to be 63 and 27 ha (Table 1). Each colony included two treatment sites and one non-treated site; each plot was separated by ≥ 150 m on Pinnacles and ≥75 m on Prairie Wind South. In this case, plot sizes varied (depending on BTPD dispersions/densities) from 0.85 to 2.06 ha (the primary objective was to evaluate flea control on BTPDs). At each colony, we evaluated two fipronil bait concentrations on separate treatment plots (0.46 and 1.52 mg fipronil/bait pellet; LaBarbera et al., 2026), with applications completed in late July (on foot using gloved hands) at 125 bait pellets/ha (and 20-m plot buffers). Trapping was completed on nearly consecutive nights for three nights just before treatments and three nights 11-15 d after treatments.

Each trap night, Sherman traps were set in the late evening, using sweet grain attractant, and checked in the early morning. We processed small mammals at trap locations or predetermined stations adjacent to the trapping grids. Herein, we concentrate on DM and GM; we also captured small numbers of hispid pocket mice (*Chaetodipus hispidus*), house mice (*M. musculus*), and voles (*Microtus* spp.). Each individual was weighed to the nearest gram with a Pesola spring balance (Baar, Switzerland). For identification purposes, we marked each small mammal with a numbered metal tag in each ear (National Band and Tag Co., Newport, KY, USA, Model 1005-1). We released all individuals at their point of capture.

### 2.3 Data analyses by field experiment

We analyzed data from each of the field experiments separately given the differences in sampling designs (BACI in 2018 and 2023, BA in 2022), sampling intensities (non-consecutive nights in 2018, consecutive or nearly consecutive nights in 2022 and 2023), and species composition (DM in all years, GM in 2022 and 2023 only). Traditional statistical testing approaches (e.g., multivariate models and *P*-values) are valuable with studies concentrating on experimental treatment effects (Burnham and Anderson, 2002). Thus, we used a frequentist approach to statistical inference. The experiments in 2018 and 2023 were of a paired design; we did not detect differences between pairs, so we analyzed those data (by year) with all treated (or non-treated) plots combined. All treated plots were combined when analyzing data from the 2022 experiment.

#### 2.3.1 Field BACI experiment in 2018

We analyzed reencounter rates for DM captured (and marked) in the period before treatment and reencountered, or not, in the period after treatment. We analyzed reencounter probabilities instead of apparent survival (that accounts for capture probability) because trapping occurred on non-consecutive nights, in some cases with 2-8 nights between trapping occasions, such that a balanced analysis was not possible at fine temporal scales. Instead, we concentrated on general trends from before to after treatment. Although this analysis does not account for imperfect capture probabilities, trapping occurred simultaneously on paired plots, at the same trap densities on all plots, such that capture probabilities should have differed little between paired plots; we investigated between-plot variation in capture probabilities with the 2022 and 2023 data and failed to detect such differences.

We analyzed the 2018 BACI field DM reencounter data in R x64 (version 4.1.2, R Core Team, 2025) using a logistic regression model (‘glm’ in ‘stats’ package) with a predictor variable for TREATMENT (non-treated, treated). Prior research, with a different bait formulation (Rocke et al., 2017), indicated bait uptake rates were higher for adult (larger) vs. juvenile (smaller) rodents on BTPD colonies (Bron et al., 2018). Therefore, we included DM body MASS (g) as a predictor variable. We also included a TREATMENT × MASS interaction; if fipronil bait pellets cause at least some DM mortality, and larger (older) DM consume more bait pellets(s), we might detect a negative effect of MASS on DM reencounters on the treated plots and a differential (perhaps positive) effect of MASS on the non-treated plots. We reduced the initial model to a parsimonious form via backward elimination (α = 0.050 for main-effects, 0.200 for interactions; ‘Anova’ in ‘car’ package; Fox et al., 2024). For interpretation, we present model predictions and standard errors (*SE*s; ‘predict’ in ‘car’ package).

Ultimately, we detected a potential negative effect of fipronil bait pellets on DM reencounter rates (for heavier individuals, in particular). Fipronil bait pellets had no detectable effect on DM abundance from before to after treatment, in this case with 30-44 d between sampling periods; we captured 37 and 40 DM on the treated plots in the before and after periods, respectively, vs. 47 and 42 DM, respectively, on the non-treated plots. We aimed to determine why DM abundance remained relatively stable from before to 30-44 d after treatment. We suspected population recruitment, including immigration and/or juvenile recruitment, were influential. We assessed population recruitment using data from the after period and a χ^2^ test to compare proportions of new captures (first detected after treatment) on the treated vs. non-treated plots.

#### 2.3.2 Field before-after experiment in 2022

In the 2022 BA experiment, trapping occurred simultaneously on consecutive nights in the before and after treatment periods. Therefore, we analyzed survival probabilities, for DM and GM combined, while accounting for capture probabilities (we did not detect interspecies differences, and the combined assessment increased statistical power). We simultaneously estimated mouse abundance, in the same models, before and after treatment while accounting for capture probabilities.

We analyzed the data under the “Robust Design with Huggins' *p* and *c*” data type in MARK (White and Burnham, 1999) to estimate survival (Φ) while accounting for imperfect capture (*p*) and recapture (*c*) probabilities. Under this model, mouse abundance ($\hat{N}$) is a derived parameter, conditioned out of the Huggins likelihood (White, 2008). Two additional parameters for movements and availability (γ′ and γ'') were estimated at ∼0 in preliminary analyses (suggesting mice that were resident before the treatments also used our study plots during at least some nights of the after period); therefore, these parameters were set to 0 in the final analysis (this reduced the number of parameters, which should have increased precision when estimating Φ, our primary objective). Each individual mouse was assigned an encounter value (1 = detected, 0 = not detected) for each trap night (secondary occasion) in the before and after treatment periods (primary occasions). We considered all possible time variations in *p* and *c* (night, period, night × period); for parsimony herein, we present results from the most supported *p* and *c* structures, with the parameters varying by period, but not varying by nights within periods. We considered an effect of mouse MASS on Φ. We selected a parsimonious model using the χ^2^ likelihood ratio test in MARK (α = 0.050). We present model predictions and *SE*s from MARK.

#### 2.3.3 Field BACI experiment in 2023

In the 2023 field BACI experiment, trapping in the before and after periods occurred simultaneously on each replicate (all three plots per BTPD colony) on consecutive or nearly consecutive nights in both periods (maximum = one night separation between trapping occasions). We analyzed the capture-recapture data as described above for the 2022 before-after experiment, with DM and GM data combined in MARK. We also combined data from the two BTPD colonies (given no inter-colony differences were detected) and concentrated primarily on the effect of TREATMENT (non-treated, low fipronil dose, high fipronil dose) and its interaction with MASS on Φ and estimates of $\hat{N}$ from the most parsimonious model. Regarding TREATMENT, we considered similar Φ on all plots, differing Φ between treated and non-treated plots, and differing Φ for all plots. We present model predictions and *SE*s from MARK.

### 2.4 Laboratory known-fate experiment in 2022

In 2022, we conducted a known-fate laboratory experiment with 33 adult DM provided by Rocky Mountain Laboratories, National Institutes of Health (NIH), National Institute of Allergy and Infectious Diseases, Hamilton, Montana, to the Animal Reproduction and Biotechnology Laboratory, Colorado State University, Fort Collins, Colorado. DM were housed individually in standard mouse cages with wood shavings and paper tissues, and water was provided ad libitum. Except when being provided a single blank acclimation pellet or experimental fipronil bait pellet, the mice were provided commercial mouse chow ad libitum (Teklad Rodent Diet, Inotiv, Inc., Lafayette, Indiana).

We weighed each DM prior to offering bait pellets to allow for indexing of forthcoming fipronil dosing. On the first evening, the mouse chow was removed, and all DM were provided a single “blank” pellet without fipronil. By the following morning, 29 (88%) had consumed their entire pellet (mouse chow was provided again to those DM). Two DM had partially consumed their pellet and two did not eat any of their pellet; these DM were not given mouse chow yet. By the next morning, the DM with prior evidence of partial or no bait consumption had consumed the remainder of their pellet; mouse chow was returned to these DM.

At the start of the experimental period, mouse chow was removed, and each DM was provided with a single bait pellet containing 0.86 mg of fipronil. The next morning, we weighed bait pellet remnants and subtracted the mass measurements from an average bait pellet mass (1.25 g) to index the percentage of bait pellet consumed by each DM, which allowed us to index fipronil doses as mg fipronil/kg body mass. We monitored the DM for mortality for three consecutive 24-hr intervals.

Each of the 33 DM was assigned an encounter history as known to be alive or dead on each day in the three 24-hr intervals of monitoring. We estimated DM survival rates using the Kaplan-Meier “known fate” model in MARK (White et al., 2001). We used χ^2^ likelihood ratio tests (α = 0.050) to determine if survival (Φ) was constant or varied by 24-hr interval, and to determine if survival was related to fipronil dose (mg fipronil/kg body mass). We also evaluated a parameter for the probability of DM surviving the duration of the 3-d period.

Fat and brain tissue samples from treated mammalian hosts can be useful in assessing fipronil residues in hosts over time posttreatment; fat tissue can be limited in DM, so we sampled brain tissues in this study. After a DM died, a sample of brain tissue was dissected and stored frozen in a glass jar or centrifuge vial. All surviving DM were euthanized in an induction chamber (CO_2_ 30-70%; American Veterinary Medical Association, 2020) on 3, 4, 5, 10, 20, 30, and 49 d post-treatment and brain tissues were collected in an effort to assess exposure. Brain tissue was available for 31 DM. Fipronil and fipronil sulfone concentrations in brain tissues were measured by validated liquid chromatography with tandem mass spectrometry at the Analytical Toxicology Laboratory, Colorado State University, Fort Collins, Colorado (Poché et al., 2020).

## 3 Results

### 3.1 Field BACI experiment in 2018

**Fig. 1:**
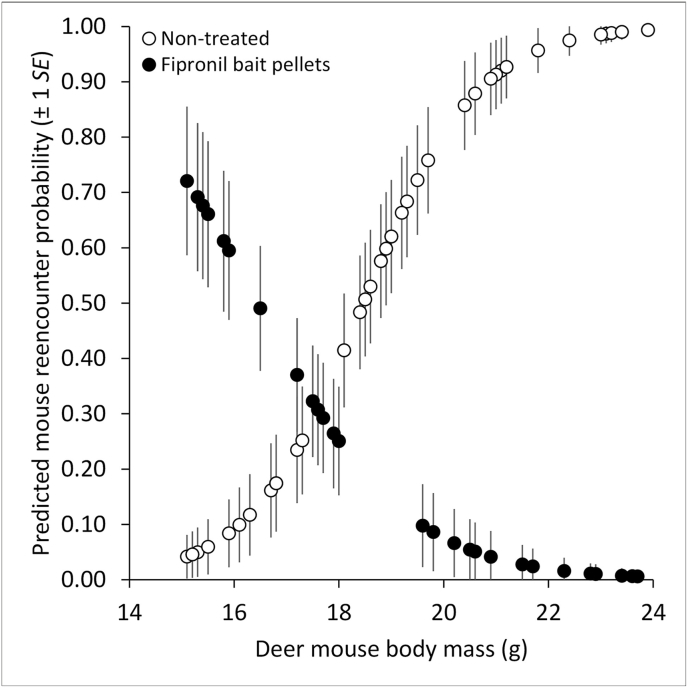
Predicted deer mouse (*Peromyscus sonoriensis*) reencounter probabilities (±1 *SE*) in the 2018 before-after-control-impact (BACI) field experiment relative to deer mouse body mass (g) and treatment (non-treated, bait pellets each with 0.84 mg fipronil/bait pellet).

The field BACI experiment in 2018 included 84 DM (37 treated plots, 47 non-treated plots) with MASS ranging from 15 to 24 g ($\bar{x}$ = 19 g). The TREATMENT × MASS interaction was supported (χ^2^ = 39.125, *P* < 0.001). On the treated plots, DM reencounter rates varied inversely with MASS; in contrast, on the non-treated plots, DM reencounter rates varied directly with MASS (Fig. 1). When accounting for MASS, DM reencounter rates from before to 30-44 d after treatment were 45% lower on the treated plots vs. non-treated plots. The proportion of new captures in the posttreatment period was 90% higher on the treated plots (73% new captures) vs. non-treated plots (38%; χ^2^ = 9.794, *P* = 0.002).

### 3.2 Field before-after experiment in 2022

**Fig. 2:**
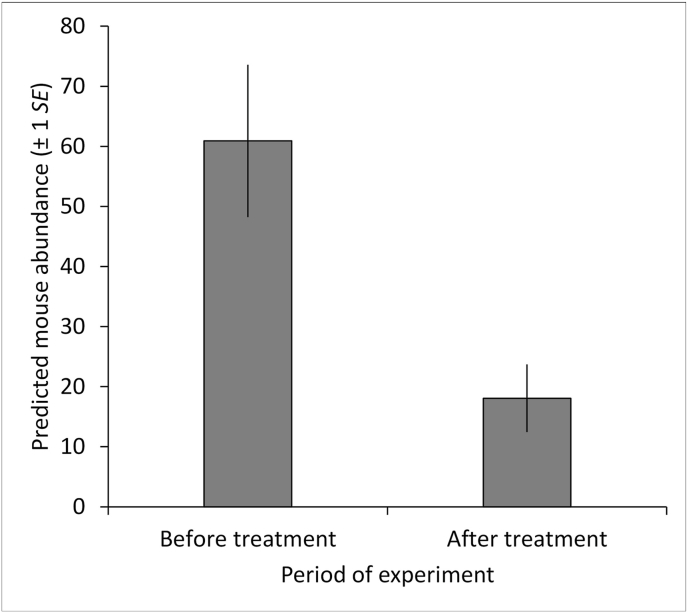
Predicted deer mouse (*Peromyscus sonoriensis*) and grasshopper mouse (*Onychomys leucogaster*) abundance combined ($\hat{N}$ ± 1 *SE*) in the 2022 before-after field experiment relative to period of the experiment (before or after treatment, with bait pellets each containing 0.96 mg fipronil/bait pellet; Eads et al., 2026).

The field before-after experiment in 2022 included 45 mice (22 DM, 23 GM), with DM MASS ranging from 14 to 24 g ($\bar{x}$ = 20 g) and GM MASS ranging from 16 to 48 g ($\bar{x}$ = 29 g; Eads et al., 2026). The most supported model included an effect of MASS (χ^2^ = 6.349, *P* = 0.012); mouse survival over 6-10 d after treatment varied inversely with MASS (similar to the results from the treated plots in 2018). Mouse survival (Φ) from before to 6-10 d after treatment was estimated at 36% (*SE* = 0.07). Mouse abundance ($\hat{N}$) declined by 70% from before to 6-10 d after treatment (Fig. 2; Eads et al., 2026).

### 3.3 Field BACI experiment in 2023

**Fig. 3:**
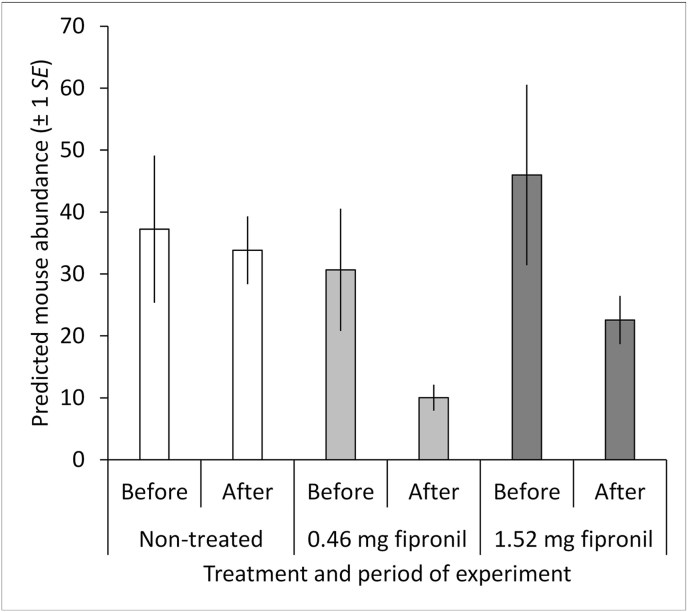
Predicted deer mouse (*Peromyscus sonoriensis*) and grasshopper mouse (*Onychomys leucogaster*) abundance ($\hat{N}$ ± 1 *SE*) in the 2023 before-after-control-impact (BACI) field experiment relative to treatment (non-treated, 0.46 mg fipronil/bait pellet, 1.52 mg fipronil/bait pellet) and period of the experiment (before or after treatment; Eads et al., 2026).

The field BACI experiment in 2023 included 89 mice (38 DM [20 non-treated, 4 high dose, 14 low dose], 51 GM [16 non-treated, 14 high dose, 21 low dose]), with DM MASS ranging from 11 to 24 g ($\bar{x}$ = 20 g) and GM MASS ranging from 11 to 46 g ($\bar{x}$ = 28 g; Eads et al., 2026). There was no statistical support for variation in survival (Φ) by fipronil dose (χ^2^ = 0.426, *P* = 0.514), for a differential effect of MASS among plots (χ^2^ = 3.380, *P* = 0.337), or for a differential effect of MASS between the non-treated and treated plots (χ^2^ = 3.237, *P* = 0.357). DM and GM survival, combined, from before to 11-15 d after treatment was 51% lower on the treated plots (Φ = 0.29, *SE* = 0.10) vs. non-treated plots (Φ = 0.59, *SE* = 0.17; χ^2^ = 4.057, *P* = 0.026). From before to after treatment, DM and GM abundance combined ($\hat{N}$) declined by 9% on the non-treated plots vs. declines of 67% and 51% on the treated plots (i.e., high and low fipronil dose, respectively; Fig. 3; Eads et al., 2026).

### 3.4 Laboratory known-fate experiment in 2022

Initial DM body masses ranged from 15 to 29 g ($\bar{x}$ = 22 g). Mass measurements from the remaining fipronil bait pelletremnants for 32 mice with such data suggested the DM consumed 5-100% of their bait pellet ($\bar{x}$ = 27%; two mice consumed an entire bait pellet). Indexed fipronil dosing ranged from 3 to 46 mg fipronil/kg body mass ($\bar{x}$ = 11 mg fipronil/kg; ∼11-12% of the LD_50_ for laboratory mice [i.e., 95 mg fipronil/kg]; Supplemental Material). Sixteen of the 33 DM died within 3 d of treatment. One additional DM died 4 days after treatment, and one died 7 days after treatment. All other DM survived to their scheduled euthanasia date.

**Fig. 4:**
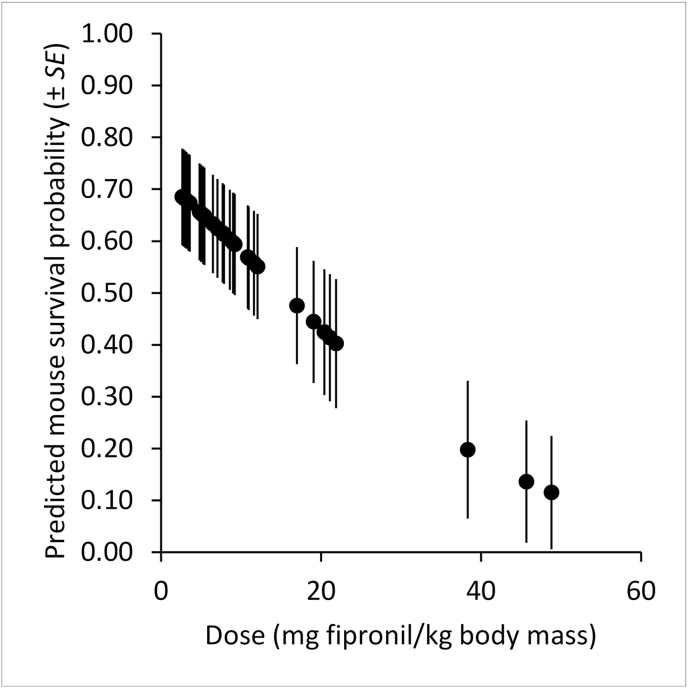
Predicted deer mouse (*Peromyscus sonoriensis*) survival probabilities (±1 *SE*) in the 2022 laboratory known-fate experiment relative to fipronil dose from a bait pellet (mg fipronil/kg deer mouse body mass; Supplemental Material).

In the analysis of DM known fates over 3 d, survival (Φ) varied by 24-hr interval (χ^2^ = 13.465, *P* = 0.001) with most deaths on day 3. DM post-treatment survival over 3 d was estimated at 47% (*SE* = 0.09; mortality = 53%). The probability of DM survival varied inversely with fipronil dose (Fig. 4; χ^2^ = 6.058, *P* = 0.014). Seven of 10 mice weighing ≤20 g $(\bar{x}$ = 17 g, range = 15-20 g) died compared to 3 of 23 mice weighing more than 20 g ($\bar{x}$ = 24 g, range = 21-29 g). Fipronil was detected in 18 of 31 DM brain samples (range = none detected to 7280 ng/g; for the samples with some detected $\bar{x}$ = 2028 ng/g; Supplemental Material). Fipronil sulfone was detected in all 31 brain samples available ($\bar{x}$ = 22,582 ng/g, range = 19-61,205 ng/g; Supplemental Material).

## 4 Discussion

### 4.1 Field experimental conditions

Among 218 mice (DM + GM) in our analyses of reencounter and apparent survival rates under natural conditions, 66% were DM, mirroring the observations of Agnew et al. (1986) on BTPD colonies at Badlands National Park in 1981-1982, with higher numbers of DM (∼70% of mice) than GM (∼30%). During our experiments, DM densities were highest in 2018 and 2022 vs. 2023. Many of the 2018 and 2022 plots were situated on portions of BTPD colonies with patches of thick vegetation during our sampling periods (namely, yellow sweet clover); DM, which prefer thick vegetative cover (Stapp and Van Horne, 1997), may have benefited and been relatively abundant for study. GM densities were higher in 2022-2023 than 2018, especially on plots with reduced vegetative cover, perhaps reflecting the affinity of GM to native forbs and bare ground (Stapp, 2007). Ultimately, the timing and placing of our field experiments provided suitable conditions for an evaluation of fipronil bait pellet effects on cricetids; sample sizes were sufficient to detect trends.

BTPD colonies are thought to be high quality habitats for GM and, perhaps, DM (Stapp, 2007), partly because BTPD burrows provide these cricetids with antipredator cover and nesting sites. Moreover, potential mouse prey items (e.g., arthropods) can be abundant on BTPD colonies (Agnew et al., 1987; Deisch et al., 1989; Sharps and Uresk, 1990). In the study of Bron et al. (2018) from 2013 to 2015 on BTPD colonies at Buffalo Gap National Grassland, north of the Badlands National Park geologic “wall”, DM and GM densities averaged 7/ha and 8/ha, respectively. In our field experiments, DM and GM densities averaged 5/ha and 1.5/ha, respectively. Among the 13 plots we sampled, 54% (i.e., 7) occurred south of the Badlands geologic wall, in the Conata Basin. Previously in 2008, a plague epizootic erupted in the Conata Basin and spread therein for several years, with few observable impacts north of the Badlands wall. Perhaps DM and GM densities were comparatively low in our study because the mouse populations, south of the wall in particular, were still suppressed by the prior epizootic, and/or plague was still festering in the area. Additional factors, such as past deltamethrin treatments (Goldberg et al., 2022) and varying precipitation may have been influential (King, 1968; Kirkland and Layne, 1989).

### 4.2 Effects of fipronil bait pellets on DM and GM reencounter and survival rates

Ideally, integrated plague management will ensure the associated tools and methods are cost-effective and sustainable, while minimizing potential adverse environmental, economic, or safety consequences for humans, wildlife, and ecosystems (CDC, 2020). We found that fipronil bait pellets, under the treatment regimens herein, are lethal to some DM and GM (Fig. 1, Fig. 2, Fig. 3, Fig. 4). The laboratory experiment indicated that even portions of a single fipronil bait pellet may be toxic to DM, with mortality observed at doses far below the reported LD_50_ of technical grade fipronil in laboratory mice (*M. musculus*; Supplemental Material). Perhaps the potential for fipronil toxicity is higher in DM and GM than *M. musculus*. Even so, laboratory studies are difficult to extrapolate to natural conditions. It remains possible that DM and GM may consume only small portions of bait pellets under natural conditions due, for example, to true avoidance or taste aversion of fipronil, or loss of appetite after the mice begin to feel sick. In the laboratory, 88% of the 33 DM consumed a complete acclimation blank pellet the first night and 100% had consumed a complete blank pellet after two nights compared to only two of the 33 DM consuming a complete fipronil bait pellet.

In 2018, DM reencounter rates from before to 30-44 d after treatment were 45% lower on the treated plots vs. non-treated plots. Yet, DM abundance remained similar from before to 30-44 d after treatment on the treated plots. In at least some cases, the *r*-selected habitat generalist DM may quickly compensate for short-term population reductions via reproduction and/or immigration. We did not capture GM in 2018; we suspect this species may have a reduced intrinsic ability to compensate for potential fipronil bait pellet induced population reductions, for instance due to possible reproductive quiescence (not observed among DM) in late fall to early winter (Pinter, 1970) and smaller litter sizes (i.e., 1-5 young/litter [Pinter, 1970] vs. ∼3-11 young/litter for DM [King, 1968; Kirkland and Layne, 1989]). Further research could enhance understanding of any population rebounds that might occur over longer spans of time for GM compared to DM.

In the 2018 (BACI) and 2022 (BA) field experiments, but not the 2023 (BACI) experiment, mouse survival on the fipronil bait pellet plots varied inversely with body mass; just the opposite of what we observed with DM in the laboratory experiment. Perhaps under natural conditions, larger mice find and consume more bait pellets (for a related example, see Bron et al., 2018), such that heavier (older) mice consume more fipronil and, in some cases, may suffer lowered survival. These mice are known to cache food so, in the wild, they may consume bait pellets over days or maybe even weeks posttreatments.

### 4.3 Scavengers

Negative effects of fipronil bait pellets on DM and GM survival may have unintended consequences. A short-term increased availability of mouse carcasses may affect scavenging species, including an obligate *Cynomys* predator (that also consumes cricetids), the highly endangered black-footed ferret (*Mustela nigripes*, hereafter ferret). If a ferret occupies a 65-ha home range (Livieri and Anderson, 2012) and DM, for instance, occur at 5/ha on a BTPD colony, and ∼50% of the DM treated with fipronil bait pellets die (as found in our laboratory experiment), the ferret might have access to ∼162 DM carcasses with varying concentrations of fipronil residues. A pulse of mouse carcasses may provide an influx of fipronil residues (a potential positive or negative outcome, depending on dosing and context).

Additional scavengers might also be considered, including PDs, other rodents, and other predators (Hoogland, 1995; Boone et al., 2009). Rattlesnakes (*Crotalus* spp.) commonly occupy PD colonies and scavenge rodent carcasses; in fact, carrion consumption is a potentially common component of snake behavior (DeVault and Krochmal, 2002). There is evidence of fipronil toxicity, even at low concentrations, in some reptile species (Tingle et al., 2003). Avian species with a tendency for scavenging might also be considered (Mctee et al., 2019). Scavenging arthropods may assist in (sometimes quickly) reducing the abundance of DM and GM carcasses (DeVault et al., 2003), but these arthropods might suffer population reductions due to the toxicity of fipronil residues in those carcasses (the binding of fipronil to insect GABA receptors is tighter than that observed for mammals; Hainzl et al., 1998; Tingle et al., 2003). Additional species that might contact fipronil bait pellets or residues in other ways may also be considered in future studies (e.g., corvids, ground dwelling birds, etc.).

### 4.4 Plague mitigation

Although we detected negative effects of fipronil bait pellets on DM and GM survival, population reductions might be short-lived, as we observed with DM in 2018, and their population dynamics may (perhaps quickly) return to prior levels after bait pellet treatments. The remaining (and recruited) DM and GM may benefit from prior treatments because flea populations are reduced (Eads et al., 2021; Matchett et al., 2023) and, presumably, *Y. pestis* transmission would be reduced (Biggins et al., 2010; Matchett et al., 2010). Over the long-term, the net effects of fipronil bait pellet treatments for DM and GM may be positive. Evidence for the net positive effect of flea control (and resulting plague mitigation) on a variety of mammals continues to accumulate (Biggins et al., 2010, 2021; Matchett et al., 2010; Goldberg et al., 2021, 2022). All that said, we were only able to evaluate posttreatment DM rebounds within a reasonable posttreatment interval in one experiment (i.e., 2018, 30-44 d posttreatment); the remaining posttreatment intervals were too short to observe any potential rebounds (2022 and 2023, 6-15 d posttreatment). Given the results of this study, continued research on potential non-target effects (e.g., Eads et al., 2025) may increase understanding.

Future research could further evaluate potential cascading treatment effects. Field experiments could be designed to determine if the results of this study scale up to larger landscapes. We conducted experiments on small plots surrounded by non-treated habitat. Additional factors could be considered including degradation of fipronil, e.g., via photodegradation and hydrolysis (Ngim et al., 2000; Raveton et al., 2006), which could influence subsequent wildlife dosing. Under some conditions, bait competition among rodents, both intra- and inter-specific, may help to mediate the effects of fipronil bait pellets on mice. Distributing bait pellets shortly after first light in the morning should maximize opportunities for PDs to find and consume them and thereby reduce the number of pellets available to small mammals that are active at night. Continued refinement of fipronil bait pellet treatments (e.g., concentrations of fipronil and application rates) may help to reduce unintended effects, while still assisting in effective flea control and plague mitigation.

## CRediT authorship contribution statement

**David A. Eads:** Conceptualization, Data curation, Formal analysis, Funding acquisition, Investigation, Methodology, Project administration, Resources, Supervision, Visualization, Writing – original draft, Writing – review & editing. **Marc R. Matchett:** Conceptualization, Data curation, Funding acquisition, Investigation, Methodology, Project administration, Resources, Supervision, Writing – original draft, Writing – review & editing. **Travis M. Livieri:** Conceptualization, Funding acquisition, Investigation, Methodology, Project administration, Resources, Supervision, Writing – review & editing. **Richard A. Bowen:** Data curation, Funding acquisition, Investigation, Methodology, Project administration, Resources, Supervision, Writing – review & editing. **Airn E. Hartwig:** Data curation, Investigation, Methodology, Project administration, Resources, Supervision, Writing – review & editing. **Stephanie Porter:** Data curation, Investigation, Methodology, Project administration, Resources, Writing – review & editing. **Mary L. Wright:** Data curation, Funding acquisition, Investigation, Methodology, Project administration, Resources, Supervision, Writing – review & editing. **Jason Fly:** Data curation, Investigation, Methodology, Project administration, Resources, Supervision, Writing – review & editing. **Madisen Hartlaub:** Data curation, Investigation, Methodology, Project administration, Resources, Supervision, Writing – review & editing. **Phillip Dobesh:** Funding acquisition, Investigation, Methodology, Project administration, Resources, Supervision, Writing – review & editing. **Paul Roghair:** Funding acquisition, Investigation, Methodology, Project administration, Resources, Supervision, Writing – review & editing. **Eddie Childers:** Funding acquisition, Investigation, Methodology, Project administration, Resources, Supervision, Writing – review & editing. **John P. Hughes:** Funding acquisition, Investigation, Methodology, Project administration, Resources, Writing – review & editing. **Michelle L. Hladik:** Data curation, Formal analysis, Funding acquisition, Investigation, Methodology, Project administration, Resources, Supervision, Writing – review & editing. **Gregory P. Dooley:** Data curation, Formal analysis, Funding acquisition, Investigation, Methodology, Project administration, Resources, Supervision, Writing – review & editing. **Brian J. Smith:** Funding acquisition, Investigation, Methodology, Project administration, Resources, Writing – review & editing. **Rachel A. LaCasse:** Funding acquisition, Investigation, Methodology, Project administration, Resources, Writing – review & editing. **Kristy Bly:** Funding acquisition, Investigation, Methodology, Resources, Writing – review & editing. **Dean E. Biggins:** Conceptualization, Funding acquisition, Investigation, Methodology, Project administration, Resources, Writing – review & editing.

## Declaration of competing interest

The authors declare that they have no known competing financial interests or personal relationships that could have appeared to influence the work reported in this paper.

## References

[bib1] Agnew W., Uresk D.W., Hansen R.M. (1986). Flora and fauna associated with prairie dog colonies and adjacent ungrazed mixed-grass prairie in western South Dakota. J. Range Manag..

[bib2] Agnew W., Uresk D.W., Hansen R.M. (1987). Arthropod consumption by small mammals on prairie dog colonies and adjacent ungrazed mixed grass prairie in western South Dakota. Great Plain. Wildlife Damage Contr. Worksh. Proc..

[bib3] American Veterinary Medical Association (2020).

[bib5] Biggins D.E., Eads D.A., Godbey J.L. (2021). Plague transforms positive effects of precipitation on prairie dogs to negative effects. Int. J. Parasitol.: Parasites Wildl..

[bib4] Biggins D.E., Godbey J.L., Gage K.L., Carter L.G., Montenieri J.A. (2010). Vector control improves survival of three species of prairie dogs (*Cynomys*) in areas considered enzootic for plague. Vector Borne Zoonotic Dis..

[bib6] Boone A., Kraft J.P., Stapp P. (2009). Scavenging by mammalian carnivores on prairie dog colonies: implications for the spread of plague. Vector Borne Zoonotic Dis..

[bib7] Bron G.M., Richgels K.L., Samuel M.D., Poje J.E., Lorenzsonn F., Matteson J.P., Boulerice J.T., Osorio J.E., Rocke T.E. (2018). Impact of sylvatic plague vaccine on non-target small rodents in grassland ecosystems. EcoHealth.

[bib8] Bron G.M., Smith S.R., Williamson J.D., Tripp D.W., Rocke T.E. (2021). Moderate susceptibility to subcutaneous plague (*Yersinia pestis*) challenge in vaccine-treated and untreated Sonoran deer mice (*Peromyscus maniculatus sonoriensis*) and northern grasshopper mice (*Onychomys leucogaster*). J. Wildl. Dis..

[bib9] Burnham K.P., Anderson D.R. (2002).

[bib10] Caro T.M. (2002). Factors affecting the small mammal community inside and outside Katavi national park, Tanzania. Biotropica.

[bib11] CDC (2020).

[bib12] Connelly P. (2011).

[bib13] Corro L.M., Tripp D.W., Stelting S.A., Miller M.W. (2017). Using off-the-shelf technologies to mass manufacture oral vaccine baits for wildlife. J. Wildl. Dis..

[bib14] Deisch M.S., Uresk D.W., Linder R.L. (1989). Ninth Great Plains Wildlife Damage Control Workshop Proceedings.

[bib16] DeVault T.L., Rhodes O.E., Shivik J.A. (2003). Scavenging by vertebrates: behavioral, ecological, and evolutionary perspectives on an important energy transfer pathway in terrestrial ecosystems. Oikos.

[bib15] DeVault T.L., Krochmal A.R. (2002). Scavenging by snakes: an examination of the literature. Herpetologica.

[bib21] Eads D., Livieri T., Matchett M.R., Childers E. (2026).

[bib18] Eads D.A., Biggins D.E., Wimsatt J., Eisen R.J., Hinnebusch B.J., Matchett M.R., Goldberg A.R., Livieri T.M., Hacker G.M., Novak M.G., Buttke D.E. (2022). Exploring and mitigating plague for one Health purposes. Curr. Trop. Med. Rep..

[bib19] Eads D.A., Livieri T.M., Dobesh P., Childers E., Noble L.E., Vasquez M.C., Biggins D.E. (2021). Fipronil pellets reduce flea abundance on black-tailed prairie dogs: potential tool for plague management and black-footed ferret conservation. J. Wildl. Dis..

[bib20] Eads D.A., Livieri T.M., Dobesh P., Hughes J.P., Fly J., Redmond H., Childers E., Schwarz M.S., Biggins D.E. (2023). Plague mitigation for prairie dog and black-footed ferret conservation: degree and duration of flea control with 0.005% fipronil grain bait. Curr. Res. Parasitol. Vector Borne Dis..

[bib22] Eads D.A., Shriner S.A., Ellis J.W., Cryan P.M., Hladik M.L., Dooley G.P., Muths E. (2025). Assessing potential collateral effects on amphibians from insecticide applications for flea control and plague mitigation. PLoS One.

[bib17] Eads D.A., Biggins D.E. (2015). Plague bacterium as a transformer species in prairie dogs and the grasslands of western North America. Conserv. Biol..

[bib23] Fox J., Weisberg S., Price B., Adler D., Bates D., Baud-Bovy G., Bolker B., Ellison S. (2024). Package 'car'. https://cran.r-project.org/web/packages/car/index.html.

[bib24] Gage K.L., Kosoy M.Y. (2005). Natural history of plague: perspectives from more than a century of research. Annu. Rev. Entomol..

[bib25] Gibbons D., Morrissey C., Mineau P. (2015). A review of the direct and indirect effects of neonicotinoids and fipronil on vertebrate wildlife. Environ. Sci. Pollut. Res. Int..

[bib26] Goldberg A.R., Biggins D.E., Ramakrishnan S., Bowser J.W., Conway C.J., Eads D.A., Wimsatt J. (2022). Deltamethrin reduces survival of non-target small mammals. Wildl. Res..

[bib27] Goldberg A.R., Conway C.J., Biggins D.E. (2021). Effects of experimental flea removal and plague vaccine treatments on survival of northern Idaho ground squirrels and two coexisting sciurids. Glob. Ecol. Conserv..

[bib28] Gunasekara A.S., Truong T., Goh K.S., Spurlock F., Tjeerdema R.S. (2007). Environmental fate and toxicology of fipronil. J. Pestic. Sci..

[bib29] Hainzl D., Cole L.M., Casida J.E. (1998). Mechanisms for selective toxicity of fipronil insecticide and its sulfone metabolite and desulfinyl photoproduct. Chem. Res. Toxicol..

[bib30] Hoogland J.L. (1995).

[bib31] Hurlbert S.H. (1984). Pseudoreplication and the design of ecological field experiments. Ecol. Monogr..

[bib32] Johnson D.H. (2002). The importance of replication in wildlife research. J. Wildl. Manag..

[bib33] King J.A. (1968).

[bib34] Kirkland G.L., Layne J.N. (1989).

[bib35] Koford C.B. (1958). Prairie dogs, whitefaces, and blue grama. Wildl. Monogr..

[bib36] Krebs C.J., Boonstra R., Gilbert S., Reid D., Kenney A.J., Hofer E.J. (2011). Density estimation for small mammals from livetrapping grids: rodents in northern Canada. J. Mammal..

[bib37] LaBarbera E.M., Sushch D., Hladik M.L., Matchett M.R. (2026).

[bib38] Livieri T.M., Anderson E.M. (2012). Black-footed ferret home ranges in Conata basin, South Dakota. West. N. Am. Nat..

[bib39] Matchett M.R., Biggins D.E., Carlson V., Powell B., Rocke T. (2010). Enzootic plague reduces black-footed ferret (*Mustela nigripes*) survival in Montana. Vector Borne Zoonotic Dis..

[bib40] Matchett M.R., Eads D.A., Cordova J., Livieri T.M., Hicks H., Biggins D.E. (2023). Flea control on prairie dogs (*Cynomys* spp.) with fipronil bait pellets: potential plague mitigation tool for rapid field application and wildlife conservation. J. Wildl. Dis..

[bib41] Mctee M., Hiller B., Ramsey P. (2019). Free lunch, may contain lead: scavenging shot small mammals. J. Wildl. Manag..

[bib42] Ngim K.K., Mabury S.A., Crosby D.G. (2000). Elucidation of fipronil photodegradation pathways. J. Agric. Food Chem..

[bib43] Pinter A.J. (1970). Reproduction and growth for two species of grasshopper mice (*Onychomys*) in the laboratory. J. Mammal..

[bib44] Poché D.M., Franckowiak G., Clarke T., Tseveenjav B., Polyakova L., Poché R.M. (2020). Efficacy of a low dose fipronil bait against blacklegged tick (*Ixodes scapularis*) larvae feeding on white-footed mice (*Peromyscus leucopus*) under laboratory conditions. Parasites Vectors.

[bib45] Poché D.M., Hartman D., Polyakova L., Poché R.M. (2017). Efficacy of a fipronil bait in reducing the number of fleas (*Oropsylla* spp.) infesting wild black‐tailed prairie dogs. J. Vector Ecol..

[bib46] R Core Team (2025). https://www.R-project.org/.

[bib47] Raveton M., Aajoud A., Willison J.C., Aouadi H., Tissut M., Ravanel P. (2006). Phototransformation of the insecticide fipronil: identification of novel photoproducts and evidence for an alternative pathway of photodegradation. Environ. Sci. Technol..

[bib48] Rocke T.E., Tripp D.W., Russell R.E., Abbott R.C., Richgels K.L., Matchett M.R., Biggins D.E., Griebel R., Schroeder G., Grassel S.M., Pipkin D.R. (2017). Sylvatic plague vaccine partially protects prairie dogs (*Cynomys* spp.) in field trials. EcoHealth.

[bib49] Sharps J.C., Uresk D.W. (1990). Ecological review of black-tailed prairie dogs and associated species in western South Dakota. Great Basin Nat..

[bib50] Singh N.S., Sharma R., Singh S.K., Singh D.K. (2021). A comprehensive review of environmental fate and degradation of fipronil and its toxic metabolites. Environ. Res..

[bib51] Smith E.P. (2002). BACI design. Encycl. Environmetr..

[bib52] Stapp P. (2007). Rodent communities in active and inactive colonies of black-tailed prairie dogs in shortgrass steppe. J. Mammal..

[bib53] Stapp P., Van Horne B. (1997). Response of deer mice (*Peromyscus maniculatus*) to shrubs in shortgrass prairie: linking small‐scale movements and the spatial distribution of individuals. Funct. Ecol..

[bib54] Tingle C.C., Rother J.A., Dewhurst C.F., Lauer S., King W.J. (2003). Fipronil: environmental fate, ecotoxicology, and human health concerns. Rev. Environ. Contam. Toxicol..

[bib55] Tripp D.W., Rocke T.E., Streich S.P., Brown N.L., Fernandez J.R.R., Miller M.W. (2014). Season and application rates affect vaccine bait consumption by prairie dogs in Colorado and Utah, USA. J. Wildl. Dis..

[bib56] Wang X., Martínez M.A., Wu Q., Ares I., Martínez-Larrañaga M.R., Anadón A., Yuan Z. (2016). Fipronil insecticide toxicology: oxidative stress and metabolism. Crit. Rev. Toxicol..

[bib57] White G.C. (2008). Closed population estimation models and their extensions in program MARK. Environ. Ecol. Stat..

[bib59] White G.C., Burnham K.P., Anderson D.R. (2001). Wildlife, Land, and People: Priorities for the 21st Century. Proceedings of the Second International Wildlife Management Congress. the Wildlife Society.

[bib58] White G.C., Burnham K.P. (1999). Program MARK: survival estimation from populations of marked animals. Bird Study.

[bib60] Zeppelini C.G., de Almeida A.M.P., Cordeiro-Estrela P. (2016). Zoonoses as ecological entities: a case review of plague. PLoS neglect. Trop. Dis..

