# Supplementary material for: Effects of fipronil bait pellets on two cricetid species: Potential implications for plague mitigation and wildlife conservation
Source: Int J Parasitol Parasites Wildl. 2026 May 22;30:101239. doi: 10.1016/j.ijppaw.2026.101239 (PMC13235477; doi:10.1016/j.ijppaw.2026.101239)
Supplement: Multimedia component 1 [file mmc1.docx]

**Effects of fipronil bait pellets on two cricetid species: Potential implications for plague mitigation and wildlife conservation**

Any use of trade, firm, or product names is for descriptive purposes only and does not imply endorsement by the U.S. Government.

**Supplemental Material**. Summary of indexed fipronil dosing and fipronil and fipronil sulfone concentrations (ng/g) in brain tissues collected from deer mice (*Peromyscus sonoriensis*) exposed to a single fipronil bait pellet (FipBit) containing 0.86 mg of fipronil during a laboratory investigation of fipronil safety under controlled laboratory conditions, 2022, Animal Reproduction and Biotechnology Laboratory, Colorado State University, Fort Collins, Colorado. We weighed each mouse (g) prior to offering bait pellets to allow for indexing of forthcoming fipronil dosing. At the start of the experimental period, each mouse was provided with a single FipBit. The next morning, we weighed FipBit remnants and subtracted the mass measurements from an average FipBit mass (1.25 g) to index the percentage of FipBit consumed by each mouse, which allowed us to index fipronil doses as mg fipronil/kg body mass. If a mouse died, a sample of brain tissue was dissected and stored frozen in a glass jar or centrifuge vial. Surviving mice were euthanized (*) in an induction chamber (CO_2_ 30-70%; AVMA 2020) on days 1, 3, 4, 5, 10, 20, 30, and 49 posttreatment and brain tissues were collected similarly. Fipronil and fipronil sulfone concentrations were measured by validated liquid chromatography with tandem mass spectrometry (Analytical Toxicology Laboratory, Colorado State University, Fort Collins, Colorado; Poché et al., 2020). Among 33 mice tested, brain samples were available from 31 mice. ND = none detected. NA = not analyzed. Please send data inquiries to David Eads ([david.a.eads@gmail.com](mailto:david.a.eads@gmail.com)).

American Veterinary Medical Association (AVMA), 2020. AVMA guidelines for the euthanasia of animals: 2020 edition. American Veterinary Medical Association, Schaumburg, Illinois, USA.

Poché, D.M., Franckowiak, G., Clarke, T., Tseveenjav, B., Polyakova, L., Poché, R.M., 2020. Efficacy of a low dose fipronil bait against blacklegged tick (*Ixodes scapularis*) larvae feeding on white-footed mice (*Peromyscus leucopus*) under laboratory conditions. Parasites & Vectors 13, 391.

|  |  |  |  | Brain tissue | |
| --- | --- | --- | --- | --- | --- |
| Mouse ID | Mass | FipBit (g) remaining | Fipronil dose (mg/kg body mass) | Fipronil (ng/g) | Sulfone (ng/g) |
| 1 | 21.00 | 0.73 | 17.01 | 4895.11 | 44440.50 |
| 2 | 25.70 | 0.46 | 21.12 | 341.82 | 48083.52 |
| 3 | 26.00 | 1.12 | 3.44 | 465.90 | 26786.34 |
| 4 | 18.80 | 0.92 | 12.06 | 241.98 | 21092.05 |
| 5 | 20.00 | 1.11 | 4.81 | 883.61 | 50877.24 |
| 6 | 15.00 | 1.01 | 10.99 | 1234.47 | 44951.21 |
| 7 | 18.00 | 0.75 | 19.08 | ND | 18735.93 |
| 8 | 23.50 | 1.07 | 5.26 | ND | 16598.22 |
| 9 | 16.50 | 0.76 | 20.40 | 7279.84 | 61205.13 |
| 10 | 27.30 | 1.04 | 5.28 | ND | 19.10 |
| 11 | 14.60 | 1.19 | 2.82 | 4985.39 | 47101.04 |
| 12 | 22.80 | 1.07 | 5.42 | 1055.84 | 33148.00 |
| 13 | 17.60 | NA | NA | 913.06 | 40023.92 |
| 14 | 18.80 | 0.00 | 45.68 | 123.12 | 34165.48 |
| 15 | 15.90 | 1.18 | 3.02 | 141.68 | 47610.24 |
| 16 | 23.20 | 1.08 | 5.03 | 6898.00 | 34247.50 |
| 17 | 16.50 | 1.08 | 7.08 | 210.78 | 40392.40 |
| 18 | 22.70 | 0.95 | 9.08 | NA | NA |
| 19* | 22.40 | 0.00 | 38.34 | 5846.67 | 19059.71 |
| 20* | 25.10 | 0.45 | 21.90 | 524.86 | 15059.14 |
| 21* | 24.80 | 0.94 | 8.59 | ND | 5965.43 |
| 22* | 22.30 | 1.00 | 7.70 | ND | 11090.76 |
| 23* | 26.00 | 0.81 | 11.63 | ND | 17316.80 |
| 24* | 20.90 | 0.92 | 10.85 | ND | 1863.58 |
| 25* | 24.40 | 1.02 | 6.48 | ND | 662.49 |
| 26* | 23.60 | 0.94 | 9.02 | ND | 264.62 |
| 27* | 28.80 | 0.92 | 7.87 | ND | 31.78 |
| 28* | 22.20 | 1.16 | 2.79 | 209.53 | 10163.07 |
| 29* | 26.00 | 1.14 | 2.91 | ND | 1454.20 |
| 30* | 21.60 | 0.96 | 9.22 | ND | 1006.67 |
| 31* | 20.60 | 1.17 | 2.67 | 260.95 | 5771.16 |
| 32* | 24.90 | 1.12 | 3.59 | ND | 856.15 |
| 33* | 21.60 | 1.15 | 3.18 | NA | NA |
